# A Novel Architecture for an Intrusion Detection System Utilizing Cross-Check Filters for In-Vehicle Networks

**DOI:** 10.3390/s24092807

**Published:** 2024-04-28

**Authors:** Hyungchul Im, Donghyeon Lee, Seongsoo Lee

**Affiliations:** Department of Intelligent Semiconductors, Soongsil University, Seoul 06978, Republic of Korea; tory@soongsil.ac.kr (H.I.); takeuser@soongsil.ac.kr (D.L.)

**Keywords:** controller area network, cybersecurity, intrusion detection system, in-vehicle network, machine learning, cross-check system

## Abstract

The Controller Area Network (CAN), widely used for vehicular communication, is vulnerable to multiple types of cyber-threats. Attackers can inject malicious messages into the CAN bus through various channels, including wireless methods, entertainment systems, and on-board diagnostic ports. Therefore, it is crucial to develop a reliable intrusion detection system (IDS) capable of effectively distinguishing between legitimate and malicious CAN messages. In this paper, we propose a novel IDS architecture aimed at enhancing the cybersecurity of CAN bus systems in vehicles. Various machine learning (ML) models have been widely used to address similar problems; however, although existing ML-based IDS are computationally efficient, they suffer from suboptimal detection performance. To mitigate this shortcoming, our architecture incorporates specially designed rule-based filters that cross-check outputs from the traditional ML-based IDS. These filters scrutinize message ID and payload data to precisely capture the unique characteristics of three distinct types of cyberattacks: DoS attacks, spoofing attacks, and fuzzy attacks. Experimental evidence demonstrates that the proposed architecture leads to a significant improvement in detection performance across all utilized ML models. Specifically, all ML-based IDS achieved an accuracy exceeding 99% for every type of attack. This achievement highlights the robustness and effectiveness of our proposed solution in detecting potential threats.

## 1. Introduction

Each year, the evolution of vehicles advances toward greater connectivity and autonomy, primarily driven by the advanced interactions among Electronic Control Units (ECUs) [1]. These ECUs are integral to controlling a wide range of vehicular functions, including engine and telematics control and the deployment of airbags. This escalating dependence on ECUs to manage vehicular technologies introduces new and complex challenges in cybersecurity [2]. ECUs are not isolated systems; they communicate with external devices through on-board diagnostics-II (OBD-II) interfaces. Additionally, in the realm of modern vehicular technology, vehicles frequently establish connections not only within themselves but with external entities. This is achieved through vehicle-to-everything (V2X) communication, which encompasses connections with other vehicles (V2V) and roadside infrastructure (V2I) [3]. These advancements, while enhancing functionality, significantly increase the potential for cybersecurity vulnerabilities, requiring continuous innovation and vigilance in vehicular technologies and security protocols. In 2021, a research team at Upstream Security released a comprehensive report on global automotive cybersecurity, analyzing 633 publicly reported incidents over the past decade. This report highlights the significant and exponential increase in cyberattacks targeting connected vehicles [4].

There are various established standards for in-vehicle communication, including the Controller Area Network (CAN), FlexRay, Local Interconnect Network (LIN), and Media Oriented Systems Transport (MOST). Of these, CAN is the most widely used protocol due to its robustness, compatibility with real-time networks, ease of maintenance, and cost-effectiveness. However, the growing number of ECUs in vehicles and their increased connectivity to external networks raise significant concerns about the lack of inherent security measures against cyber-threats. Smith et al. [5] initially highlighted the fundamental security vulnerabilities of CAN. Further, Aliwa et al. [2] extensively explored these vulnerabilities through a variety of physical and remote access attacks. These vulnerabilities substantially increase cybersecurity risks, highlighting the critical need for enhanced protective measures within vehicle communication systems. Consequently, automotive manufacturers face the critical task of either eliminating malicious entities or effectively detecting and blocking malicious messages to prevent potential dangers.

**Figure 1:**
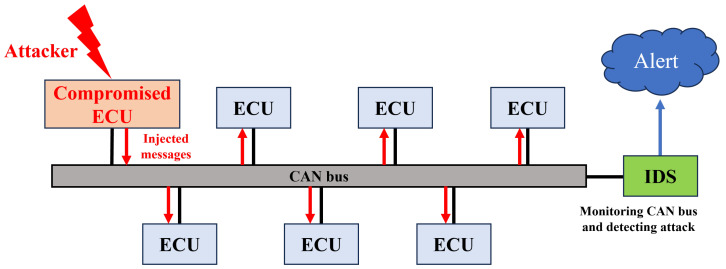
Typical structure of an intrusion detection system for the CAN bus.

To address these challenges, numerous studies have focused on using intrusion detection systems (IDS) to detect malicious messages. As shown in Figure 1, all messages transmitted via the CAN bus are monitored by these systems. Machine learning (ML)-based methods have attracted particular attention due to their effectiveness in identifying such threats. For instance, Yang et al. [6] employed tree-based classifiers, while Martinelli et al. [7] utilized a fuzzy nearest-neighbor algorithm. Additionally, Avatefipour et al. [8] proposed an anomaly detection model based on a one-class Support Vector Machine. In 2021, Moulahi et al. [9] evaluated the performance of conventional ML-based IDS systems, such as Decision Trees (DT), Random Forests (RF), Multilayer Perceptrons (MLP), and Support Vector Machines (SVM). Deep learning (DL) techniques have been proposed as well, as demonstrated by Song et al. [10], who focused on advanced architectures. In another example, Wei et al. [11] proposed a domain adversarial neural network-based IDS capable of detecting various types of attacks. Similarly, Lo et al. [12] introduced HyDL-IDS, which combines CNN and LSTM structures. Salek et al. [13] explored the use of a classical neural network and a quantum restricted Boltzmann machine (RBM).

Regardless of whether they rely on DL or ML architectures, existing IDS systems primarily depend on a single model inference to detect attacks. However, systems that directly impact human safety, such as vehicular security, require procedures that can recognize uncertain predictions and make corrections. Moreover, existing IDS systems often neglect the actual attack patterns observed in CAN bus traffic. For example, historical statistics of CAN data frames, which include both message IDs and payload data, are valuable inputs for an IDS. However, these data are rarely utilized in traditional systems. Therefore, the efficacy of intrusion detection can be substantially improved by implementing straightforward rules derived from the history of CAN bus attack patterns and incorporating them into existing IDS.

This study presents a novel IDS architecture that incorporates simple rule-based filters to validate the results generated by a conventional ML-based IDS. Considering that the proposed IDS principally relies on traditional ML techniques, it remains sufficiently lightweight for implementation in in-vehicle edge computing devices [14,15]. In addition, these rule-based filters are computationally inexpensive and can be seamlessly integrated into nearly all existing ML-based IDS frameworks. To the best of our knowledge, this topic has not yet been considered in the literature on in-vehicle networks. The results of our experimental study revealed a significant enhancement in detection performance across various traditional ML-based IDS, including RF, k-Nearest Neighbors (kNN), MLP, and SVM.

The main contributions of this study are as follows:

1. This paper highlights the importance of cross-checks for IDS predictions and proposes a novel architecture that verifies and revises these predictions.
2. The proposed novel architecture is specifically designed for intrusion detection systems in vehicles, focuses on enhancing the cybersecurity of the CAN bus, and includes specially designed rule-based filters.
3. The rule-based filters work alongside conventional ML-based IDS to cross-check predictions, thereby addressing the low detection performance commonly encountered in existing systems.

The rest of this paper is organized as follows. Section 2 introduces related work. In Section 3, we provide background information on the CAN bus and various attack types. The proposed architecture is presented in Section 4. Section 5 presents the experimental results and discussion. Finally, Section 6 concludes the study and describes future research.

## 2. Related Works

Over the past few years, intrusion detection in the CAN bus context has attracted significant attention from researchers. This surge in interest is largely attributed to fundamental security vulnerabilities inherent in CAN communication protocols. Consequently, there has been a substantial increase in academic research in this field. The literature on this topic is extensive, and encompasses a range of studies, analyses, and reviews. This is evident from several comprehensive surveys and research papers focusing on the CAN IDS, as cited in [2,16,17,18,19,20]. These studies collectively highlight the growing importance of developing robust security solutions for the CAN bus, reflecting the broader push within the automotive industry to ensure cybersecurity in modern vehicles.

Song et al. [21] proposed a streamlined method to detect intrusions in network systems by leveraging the timing intervals of message transmissions. Subsequently, an in-depth study was conducted that focused on the frequency domain characteristics of timing intervals in CAN messages [22]. The proposed method does not require any modifications to the existing CAN bus, and is designed for efficient execution on platforms with extremely limited computational resources. Similarly, Lee et al. [23] proposed a method for analyzing the ratio of the offset and time difference between sending and receiving messages which focuses on the remote and data frames. By assessing the response behaviors of ECUs, this method can distinguish between normal operations and potential security breaches, such as malicious messages. However, the study did not provide a crucial metric, such as the accuracy of attack detection, which is essential for evaluating the effectiveness of the proposed method.

Recently, innovative methods that utilize deep neural networks have been proposed. Kang and Kang [24] proposed a Deep Belief Network (DBN)-based intrusion detection to distinguish between normal behavior and attacks. A comparative analysis revealed that their DBN-based IDS outperformed traditional neural networks, particularly in enhancing detection precision while maintaining real-time responsiveness. However, the process of training a DBN is time-consuming, resulting in significant overhead. Song et al. [10] proposed an IDS using a deep convolutional neural network (DCNN). To train the DCNN model, arbitration IDs were extracted from CAN packets. These IDs, initially in hexadecimal format, were converted into binary IDs. Subsequently, binary versions of 29 sequential arbitration IDs were used to generate an image with dimensions of 29 × 29. This approach effectively led to the accurate classification of the majority of attack and normal packets into their respective categories. However, Desta et al. [25] observed that DCNN-based IDS could only distinguish between attack and normal packets when there a high volume of attack packets is present within a short time frame. Hence, they proposed a new method utilizing recurrence plots called Rec-CNN based IDS. Their approach showed enhanced effectiveness in scenarios with fewer attack packets over a longer duration. Gao et al. [26] proposed CanNet, a lightweight model that utilizes a specially designed CAN image generation scheme to convert CAN traffic data into images, which is suitable for DoS attacks.

The supervised learning-based IDS approach depends on a substantial volume of fully annotated data during the training phase of the model. In addition, these models have fundamental limitations in detecting unknown attacks. To solve these problems, Seo et al. [27] pioneered the application of a Generative Adversarial Network (GAN) model, which is an unsupervised learning approach, for intrusion detection in a CAN bus. The method was designed to learn the operational patterns of CAN traffic and identify attacks by spotting deviations from the baseline behavior. They detected each of four attacks, with an average accuracy of 98%. However, when compared to supervised learning-based IDS, the performance was lower, though GIDS remained efficient, even in contemporary contexts where there is a deficiency in establishing known attack patterns for vehicles. Xie et al. [28] proposed an enhanced GAN-based IDS to overcome the limitation of generating imprecise CAN message blocks. Agrawal et al. [29] proposed an IDS that employs Long Short-Term Memory (LSTM) combined with reconstruction and thresholding techniques to identify various attacks such as DoS, fuzzy attacks, RPM spoofing, and gear spoofing. Furthermore, Araujo-Filho et al. [30] presented an intrusion prevention system (IPS) for the CAN bus using an isolation forest (iForest), which not only detects malicious messages but discards them as well.

Deng et al. [31] noted that general ML/DL models differ from human students, who often double-check their answers during examinations, especially when their confidence is low. This is because ML/DL models typically produce an answer with a single inference. Therefore, they proposed a double-check framework that recognizes unreliable predictions and revises them. Similarly, existing IDS systems for vehicular security rely only on a single inference of IDS. In this context, we use rule-based filters to identify unreliable predictions. These filters assume that unreliable predictions result from incorrect pattern recognition by the IDS. Furthermore, they revise predictions based on the nature of the attacks, for example, ’Does the frame have enough low IDs to dominate the CAN bus given the function of a DoS attack?’

## 3. Theoretical Background

### 3.1. Controller Area Network

**Figure 2:**
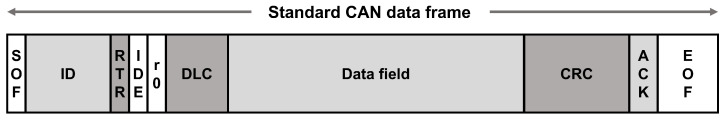
Structure of the standard CAN data frame.

The Controller Area Network (CAN) bus is a serial communication protocol engineered for real-time control systems in vehicles [32]. The majority of sensors, actuators, and processors within a vehicle communicate through this network, which employs twisted differential-pair lines to enhance noise and error resilience. The CAN message format is designed based on two criteria: the standard frame, as defined by CAN 2.0A, and the extended frame, outlined in CAN 2.0B. Figure 2 shows the standard CAN data frame. The CAN bus operates on two voltage levels, termed “dominant” (corresponding to a digital “0”) and “recessive” (corresponding to a digital “1”). In situations where multiple nodes attempt simultaneous transmission of conflicting signals, the “dominant” voltage level prevails, ensuring that a digital “0” supersedes a digital “1”. Importantly, all the nodes in this network function as masters, permitting any node to initiate a data transmission when the bus is idle. During the arbitration phase, the bits of this identifier are compared and resolved from the most significant bit (MSB) to the least significant bit (LSB). Specifically, nodes transmitting a “0” continue their transmission while those transmitting a “1” switch to a receiving mode. This mechanism ensures that the CAN node with the lowest message ID invariably completes transmission without interruption.

### 3.2. Attack Model

Common existing CAN bus attacks are categorised into denial-of-service (DoS), spoofing, and fuzzy attacks based on the level of insight the attacker has into the bus system being targeted, the intention behind the attack, and the technique being used. These categories are elaborated as below.

#### 3.2.1. DoS Attack

**Figure 3:**
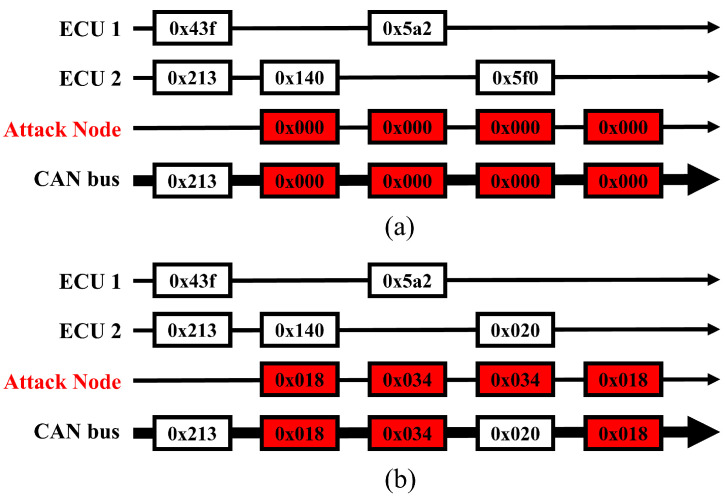
DoS attack scenario in CAN bus: (**a**) simplest DoS and (**b**) advanced DoS.

During a DoS attack on a CAN bus, the attacking node aggressively dominates the communication bus. This is achieved by sending frames with exceptionally high priority, typically represented by a message ID of 0x000 or another value that is comparably low, as shown in Figure 3. The simplest DoS attack type is performed using the highest priority ID, as shown in Figure 3a. Park et al. [33] introduced an advanced DoS attack, shown in Figure 3b, which has a sufficiently high message ID to dominate the CAN bus. This attack method is designed to exploit the priority-based arbitration of the CAN protocol, in which lower message IDs have higher priority. In this way, the attacker can launch a DoS attack even with minimal pre-existing knowledge.

The nature of this attack is crucial when designing defense mechanisms such as rule-based filters in an IDS. For example, if the IDS incorrectly classifies a CAN frame with a high message ID as a DoS attack, the filter should recognize this wrong prediction and revise it. Therefore, considering the subtleties of DoS attacks on the CAN bus, it is crucial for an IDS to differentiate between genuine high-priority messages and those used maliciously during an attack. A sophisticated rule-based filter must consider the context and frequency of these high-priority messages in order to make an accurate assessment. This understanding forms the basis for the development and refinement of more effective and nuanced intrusion detection strategies within CAN networks.

#### 3.2.2. Spoofing Attack

**Figure 4:**
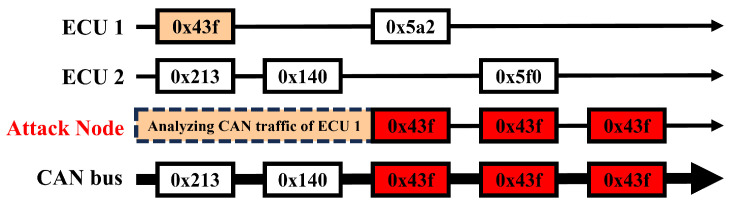
Spoofing attack scenario in CAN bus.

In spoofing attacks targeting the CAN bus, the assailant undertakes a thorough preliminary analysis of the CAN traffic, as shown in Figure 4. This pre-attack phase involves the close monitoring and recording of bus traffic, with a particular focus on identifying message IDs that correspond to the functionalities of different subsystems. Consequently, in order to execute a spoofing attack the attacker must possess a detailed understanding of the CAN bus operation. When the attack commences, the attacker continuously transmits data frames with the same payload, inducing system malfunctions.

Recognizing the nuanced nature of such attacks, the development of rule-based filters for IDS must consider these characteristics. An essential component of such a filter is the incorporation of comprehensive statistical analysis. This analysis should focus on historical data concerning the frequency and patterns of specific message IDs and payloads, particularly those that have been previously identified as attack indicators [34]. By meticulously evaluating these historical data, the filter can be fine-tuned to more accurately discern between normal operations and potential spoofing attacks.

#### 3.2.3. Fuzzy Attack

**Figure 5:**
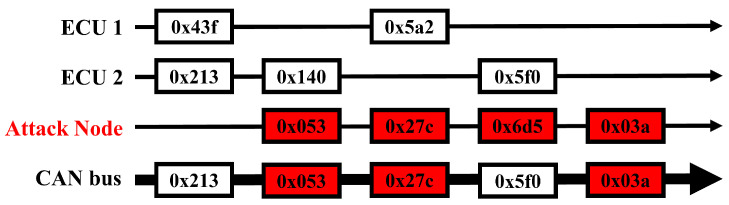
Fuzzy attack scenario in CAN bus.

In the case of a fuzzy attack on the CAN bus, the approach adopted by a malicious node is notably distinct and presents unique challenges for detection. In these attacks, a node disrupts the network by sending intentionally randomized data frames, as shown in Figure 5. These frames contain message IDs and payloads that do not follow predictable or consistent patterns; therefore, the attacker can launch this type of attack without in-depth knowledge of CAN bus operations, much like a DoS attack.

Despite this randomness, a critical aspect that can be leveraged for detection is the distinct difference between the characteristics of these frames and those of normal network traffic. In a standard operating environment, message IDs and payloads follow a certain structure and frequency that is consistent with the functional requirements of the network. In contrast, the frames generated in a fuzzy attack have message IDs and payloads that starkly contrast with this normative pattern. Such distinctiveness is a pivotal consideration for the proposed rule-based filter, and the statistics pertaining to message IDs and payloads from prior normal frames should be rigorously evaluated in the final classification.

## 4. Proposed Architecture

**Figure 6:**
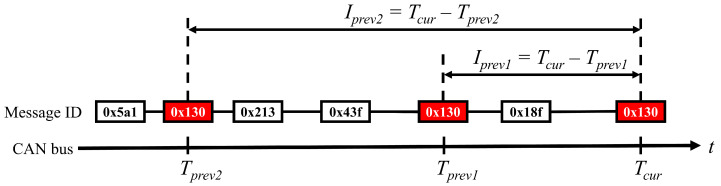
Extracting time intervals between the same CAN identifiers on the CAN bus for training.

In instances of vehicular hacking, attack frames are surreptitiously injected into the CAN bus of a vehicle. As a consequence of this signal injection, alterations occur in the sequential pattern of CAN IDs [10,21,22,25]. Accordingly, in this study, ML-based IDS variants were trained using temporal intervals associated with identical CAN IDs, as shown in Figure 6. Specifically, two temporal intervals, $I_{prev1}=T_{cur}-T_{prev1}$, $I_{prev2}=T_{cur}-T_{prev2}$, were employed as features for training. Here, $T_{cur},T_{prev1},$ and $T_{prev2}$ denote the timestamps of the current frame, immediate prior frame with the same message ID, and penultimate frame with the same message ID, respectively. It is important to note that $I_{prev1}<I_{prev2}$.

**Figure 7:**
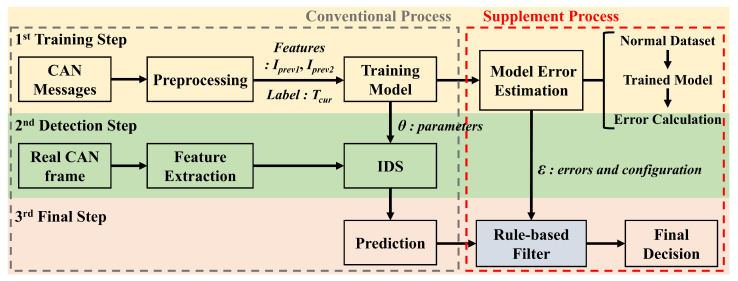
The architecture of the intrusion detection system supplemented with a cross-check rule-based filter.

Figure 7 shows the proposed architecture of IDS with cross-check rule-based filter. Typically, an IDS consists of a training step and a detection step. However, the proposed architecture includes an additional final step following the detection step, resulting in a total of three phases. In the training step, two intervals $I_{prev1}$ and $I_{prev2}$ are used as inputs, and labeling is based on $T_{cur}$. This labeling method enables real-time detection, as the IDS predicts whether an attack is occurring based on the current frame. After the training process, the trained model is used as the IDS. The real CAN frames without labels are used in the detection step to extract time intervals. Therefore, the IDS is considered a function that takes two intervals as input and outputs the label of this input. The function can be expressed as

(1) Y=f(X,θ),

where *Y* is the prediction of the IDS, *X* represents the input data, θ means the parameters of the IDS, and *f* represents the IDS. In the final step, prediction outcomes from the IDS trained with two intervals are subjected to verification through an additional rule-based filter. The final decision can be formulated in a similar way to the expression of Equation (1), provided by

(2) $$Y^{\prime }=g(Y,\varepsilon ),$$

where $Y^{\prime }$ is the final label and is classified as either attack or normal, ε denotes the IDS error and normal CAN data configuration, and *g* represents the rule-based filter. This filter is meticulously developed to take full advantage of the unique characteristics presented by the three attack scenarios outlined previously.

- **DoS attack:** This attack type obstructs the transmission of messages with low-priority identifiers by flooding the CAN bus with high-priority message IDs.
- **Spoofing attack:** Attackers can manipulate vehicle subsystems by sending a large number of CAN frames, each with a given constant but valid value.
- **Fuzzy attack:** The message IDs in a fuzzy attack are randomly selected, and the data field bits are randomly generated.

The operational principles of the proposed rule-based filter are as follows (Section 4.1, Section 4.2, Section 4.3).

### 4.1. Denial-of-Service Attack Scenario

The proposed rule-based filter executes the following protocols to verify the classification results generated by a conventional IDS (Algorithm 1).
**Algorithm 1** DoS Attack Scenario1:Train the ML-based IDS model2:**Input**: Features {$I_{prev1}$, $I_{prev2}$}3:**Output**: Final Decision {‘attack’ or ‘normal’}4:**while** Monitoring results of the IDS **do**5:    **if** $T_{cur}-T_{update}>T_{reset}$ **then**6:        $AC[ID_{cur}]\leftarrow 0$7:        $T_{update}\leftarrow T_{cur}$8:    **end if**9:    **if** decision of the IDS is DoS Attack **then**10:        $AC\left[ID_{cur}\right]\leftarrow AC\left[ID_{cur}\right]+1$11:        **if** $AC\left[ID_{cur}\right]>FP_{max}$ **then**              ▹(Rule 1)12:           attack←True13:        **else if** $ID_{cur}>ID_{th}$ **then**               ▹(Rule 2)14:           attack←False15:        **else**16:           attack←True17:        **end if**18:    **else**19:        **if** $AC\left[ID_{cur}\right]>FP_{max}$ **then**              ▹(Rule 1)20:           attack←True21:        **else**22:           attack←False23:        **end if**24:    **end if**25:**end while**

#### 4.1.1. DoS Attack: First Rule

**(Rule 1) A current frame shall be classified as an attack, irrespective of the initial judgment by the IDS, if its message ID has been recurrently categorized as an attack by the IDS which has been trained with intervals between same message IDs.**

For each individual message ID, the attack count, denoted as AC[ID], is incremented whenever the IDS classifies a corresponding frame as an attack. When the message ID for the current frame is $ID_{cur}$, its associated attack count $AC[ID_{cur}]$ is evaluated against a threshold $FP_{max}$. The attack count is reset after a certain period $T_{reset}$ to prevent the continuous increase of $AC[ID_{cur}]$ and avoid the generation of false positives (FP). We propose that if $T_{cur}-T_{update}>T_{reset}$, then the attack count is reset; here, $T_{cur}$ and $T_{update}$ respectively represent the current time and the time when the monitoring of CAN communication began, $T_{reset}$ indicates the total duration of normal CAN communication in the normal dataset, and $T_{update}$ is replaced with $T_{cur}$ after $T_{reset}$.

$FP_{max}$ represents the highest value of FP[ID] recorded across all message IDs. Following the training phase, which utilizes a DoS attack dataset, the IDS is evaluated using a normal dataset. Here, FP[ID] denotes the number of FP cases recorded during the testing phase. Consequently, $FP_{max}$ represents the most egregious classification error committed by the IDS. It is imperative to recognize that when $AC\left[ID_{cur}\right]>FP_{max}$, the attack count corresponding to the CAN ID of the current frame exceeds the inherent error margin of the IDS. Under such circumstances, the frame should be designated as an attack regardless of the initial IDS assessment.

#### 4.1.2. DoS Attack: Second Rule

**(Rule 2) A current frame shall be classified as normal, irrespective of the IDS’s preliminary assessment, if its current message ID surpasses a predetermined threshold.**

In this study, we propose using the predetermined threshold $ID_{th}$ to cross-check the predictions of the IDS. The actual IDs and data of a vehicle’s CAN messages depend on the Database Container (DBC), which is proprietary to the automotive manufacturer [35]. The best method is to access the DBC in order to determine the appropriate $ID_{th}$. However, for security reasons, the DBC file is kept strictly by the manufacturer. Thus, we analyze a normal dataset and determine the $ID_{th}$ according to the experimental results in Section 5.

During a DoS attack, the attacking node typically sends out frames with high priority, which correspond to lower message IDs in the context of the arbitration phase in the CAN bus. In such scenarios, if the current message ID, denoted as $ID_{cur}$, surpasses $ID_{th}$, then there exists a strong likelihood that the frame is not part of a DoS attack but rather a normal communication. This understanding is based on the knowledge that frames with higher message IDs face challenges in dominating the CAN bus traffic. Attack frames with high message IDs struggle to gain control over the bus because of their lower priority, which is the opposite of what occurs during DoS attacks. This behavioral pattern is a key observation for distinguishing between normal operations and potential security breaches in CAN traffic.

### 4.2. Spoofing Attack Scenario

The proposed rule-based filter executes the following protocols to verify the classification results of a conventional IDS (Algorithm 2).

#### 4.2.1. Spoofing Attack: First Rule

**(Rule 1) A frame shall be classified as an attack, irrespective of the initial IDS classification, if both its current message ID and payload data have been excessively categorized as attacks.**

In the context of a spoofing attack, Rule 1 parallels its counterpart in the DoS attack scenario; however, in the spoofing attack context, both the message ID and payload data are taken into account. Yu et al. [36] conducted an analysis of CAN communication data, identifying the transmission frequency and data variation range for each ECU device; for example, the attacker needs to send a large number of malicious messages, each with the same data field and a valid ID, within a short period of time in order to cause a malfunction.

For each distinct message ID, an ID attack count $AC_{I}\left[ID\right]$ is incremented when the IDS designates a frame as an attack. Correspondingly, for each unique payload datum, the payload data attack count $AC_{D}\left[DATA\right]$ increases. When the current message ID and payload data are denoted as $ID_{cur}$ and $Data_{cur}$, their respective attack counts $AC_{I}\left[ID_{cur}\right]$ and $AC_{D}\left[DATA_{cur}\right]$ are contrasted with predetermined thresholds $FP_{I,max}$ and $FP_{D,max}$. Therefore, in a spoofing attack scenario, both the ID attack count and the payload data attack count are reset after $T_{reset}$.

Here, the variables $FP_{I,max}$ and $FP_{D,max}$ represent the highest values of FP[ID] and FP[DATA] across all the message IDs and payload data, respectively. Following training of the IDS on a spoofing attack dataset, its performance is assessed using a normal dataset. In this instance, FP[ID] and FP[DATA] signify the number of FP cases identified in the test results for each individual message ID and payload. Consequently, $FP_{I,max}$ and $FP_{D,max}$ represent the maximum errors engendered by the IDS. If $AC_{I}\left[ID_{cur}\right]>FP_{I,max}$ and $AC_{D}\left[DATA_{cur}\right]>FP_{D,max}$, then the frame in question should be designated as an attack, disregarding the initial IDS classification.
**Algorithm 2** Spoofing Attack Scenario1:Train the ML-based IDS model2:**Input**: Features {$I_{prev1}$, $I_{prev2}$}3:**Output**: Final Decision {‘attack’ or ‘normal’}4:**while** Monitoring results of the IDS **do**5:    **if** $T_{cur}-T_{update}>T_{reset}$ **then**6:        $AC_{I}\left[ID_{cur}\right]\leftarrow 0$7:        $AC_{D}\left[DATA_{cur}\right]\leftarrow 0$8:        $T_{update}\leftarrow T_{cur}$9:    **end if**10:    **if** decision of the IDS is Spoofing Attack **then**11:        $AC_{I}\left[ID_{cur}\right]\leftarrow AC_{I}\left[ID_{cur}\right]+1$12:        $AC_{D}\left[DATA_{cur}\right]\leftarrow AC_{D}\left[DATA_{cur}\right]+1$13:        **if** $AC_{I}\left[ID_{cur}\right]>FP_{I,max}$ **and**              ▹(Rule 1)          $AC_{D}\left[DATA_{cur}\right]>FP_{D,max}$ **then**14:           attack←True15:        **else if** $AC_{I}\left[ID_{cur}\right]>FP_{I,max}$ **and**            ▹(Rule 2)         $AC_{D}\left[DATA_{cur}\right]\leq FP_{D,max}$ **then**16:           attack←False17:        **else**18:           attack←True19:        **end if**20:    **else**21:        **if** $AC_{I}\left[ID_{cur}\right]>FP_{I,max}$ **and**              ▹(Rule 1)         $AC_{D}\left[DATA_{cur}\right]>FP_{D,max}$ **then**22:           attack←True23:        **else**24:           attack←False25:        **end if**26:    **end if**27:**end while**

#### 4.2.2. Spoofing Attack: Second Rule

**(Rule 2) A frame shall be classified as normal, even if the IDS initially classifies it as an attack, under the condition that the current message ID has been repeatedly classified as an attack but the current payload data have not.**

This rule specifies that if a frame’s current message ID has been frequently classified as an attack but the payload data have not been similarly classified, then the frame should be considered normal. This is because if $AC_{I}\left[ID_{cur}\right]$ exceeds $FP_{I,max}$ within $T_{reset}$, then it can be inferred that an attack involving CAN frames with $ID_{cur}$ has occurred. However, it is essential to distinguish these from normal frames that have the same ID. Typically, spoofing attacks continuously inject frames with identical payload data in order to disrupt specific devices [28]. This guideline serves as an additional layer of verification to reduce FPs.

In a spoofing attack, the attacker deliberately transmits frames with a specific message ID corresponding to the targeted subsystem, thereby maintaining uniformity in the payload data. This behavior contrasts with the operations of a normal node, which slightly varies the payload data while keeping the message ID constant [34,36,37]. Considering these characteristics, the frame should be classified as normal if the IDS initially designates it as an attack but the payload data have not been frequently classified as part of an attack.

### 4.3. Fuzzy Attack Scenario

The proposed rule-based filter executes the following protocols to verify the classification results of a conventional IDS (Algorithm 3).
**Algorithm 3**  Fuzzy Attack Scenario1:Train the ML-based IDS model2:**Input**: Features {$I_{prev1}$, $I_{prev2}$}3:**Output**: Final Decision {‘attack’ or ‘normal’}4:**while** Monitoring results of the IDS **do**5:    **if** decision of the IDS is Fuzzy Attack **then**6:        attack←True7:    **else**8:        **if** $ID_{cur}\notin Z_{normal}$ **then**                  ▹(Rule 1)9:           attack←True10:        **else if** $H_{cur}>H_{max}\left[ID_{cur}\right]$ **and**             ▹(Rule 2)        $BC_{cur}\geq DLC_{cur}/2$ **then**11:           attack←True12:        **else**13:           attack←False14:        **end if**15:    **end if**16:**end while**

#### 4.3.1. Fuzzy Attack: First Rule

**(Rule 1) A frame shall be classified as an attack, irrespective of the IDS’s initial classification, if the current message ID of the frame is absent in the normal dataset.**

The set of all message IDs in the normal dataset, designated as $Z_{normal}$, forms the basis for vehicular control within the CAN bus. This dataset reflects the standard message flow as defined by the vehicle’s Database Container (DBC). In situations where access to the proprietary DBC file is restricted for security reasons (a common practice among automotive manufacturers), the reliance on $Z_{normal}$ becomes crucial. This is particularly the case in scenarios where direct access to the DBC is unavailable, making the normal dataset essential for identifying deviations. Therefore, if the current message ID $ID_{cur}$ is not found in $Z_{normal}$, it is regarded as an unfamiliar ID, suggesting that it does not belong to the standard message set and potentially indicating an attack. Similarly, Olufowobi et al. [38] proposed the use of a lookup table that contains a list of all message IDs to detect attack frames.

#### 4.3.2. Fuzzy Attack: Second Rule

**(Rule 2) A frame shall be classified as an attack, irrespective of the IDS’s initial classification, if it satisfies two specific conditions concerning its payload data:**

- **Condition (1): The Hamming distance [39] between the payload data of the current frame and its predecessor surpasses any such distance observed between consecutive frames with the identical message ID in the normal dataset.**
- **Condition (2): A majority of the bytes in the current payload data contains a higher number of “1” bits than any corresponding byte positions in the payload data of the normal dataset.**

In the context of a fuzzy attack, payload data are arbitrarily generated, and diverge significantly from the payload data in normal frames. Two primary divergences can be identified: the Hamming distance between consecutive frames **Condition (1)**, and the number of “1” bits at corresponding byte positions **Condition (2)**. Therefore, if both conditions are satisfied, the frame is classified as an attack.

**Figure 8:**
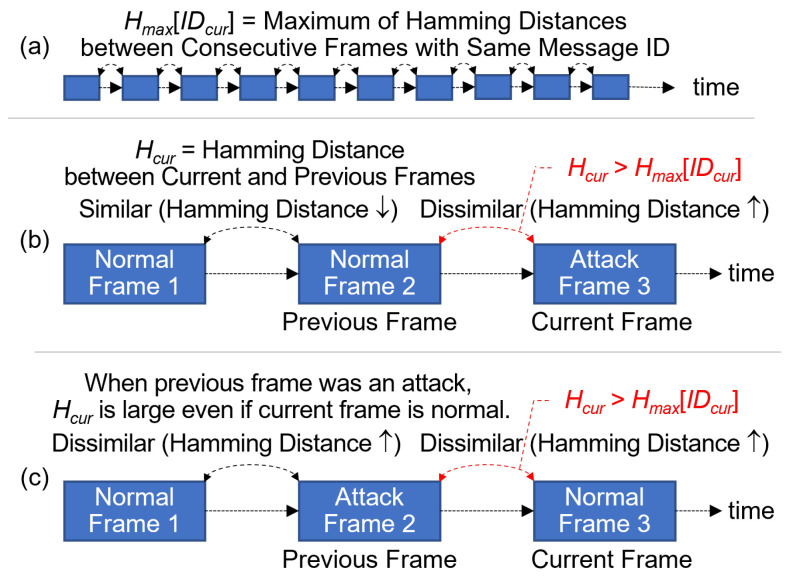
Hamming distance between two consecutive payloads with the same message ID. **(a) searching for $H_{max}$ between consecutive frames with the same Message ID; (b) current frame attack detection by Hamming distance exceeding $H_{max}$; (c) normal frame exhibiting Hamming distance exceeding $H_{max}$.**

Figure 8 shows **Condition (1)**, which assesses the dissimilarity between the payload data of two consecutive frames with the same message ID. In the case of normal frames, consecutive payloads exhibit a high degree of similarity. Conversely, payloads differ significantly between normal and attack frames [40].

In Figure 8a, $H_{cur}$ represents the Hamming distance between the payload data of the current frame and its immediate predecessor, both of which share the same message ID, denoted as $ID_{cur}$. The variable $H_{max}\left[ID_{cur}\right]$ signifies the maximum Hamming distance recorded between any two consecutive frames in the normal dataset that share the same message ID $ID_{cur}$. In Figure 8b, the inequality $H_{cur}>H_{max}\left[ID_{cur}\right]$ holds true when the current frame is under attack; however, Figure 8c presents a complicating factor in that the same inequality $H_{cur}>H_{max}\left[ID_{cur}\right]$ holds true when the current frame is actually normal. This overlap in conditions necessitates additional parameters for accurate differentiation between normal and attack frames.

**Figure 9:**
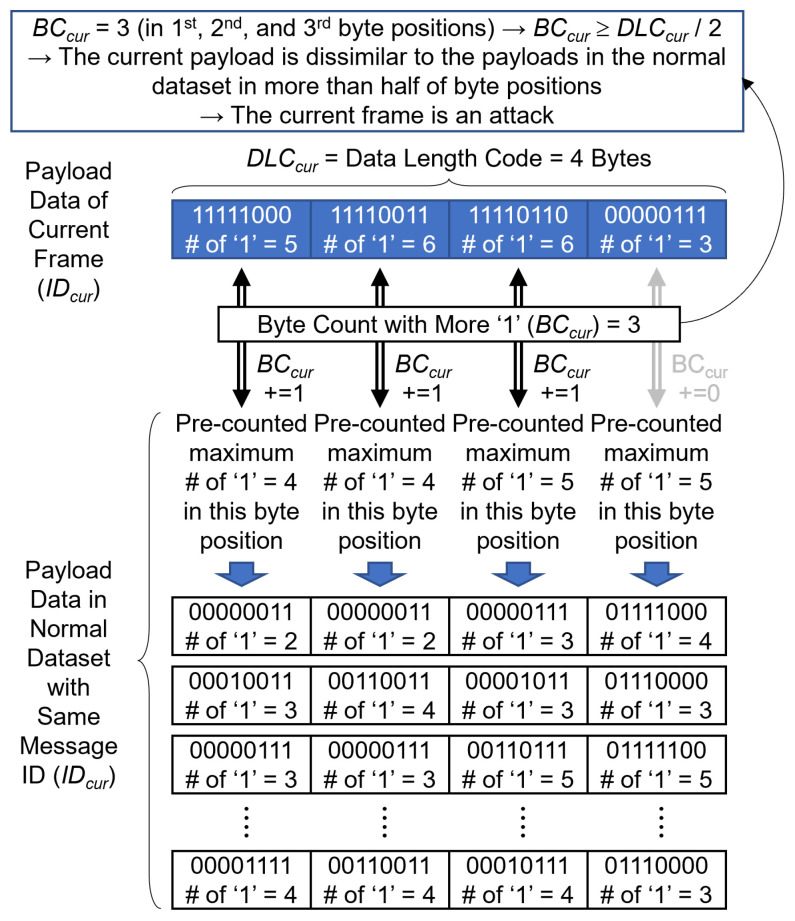
Method for byte count based on dissimilarity of payload data in byte positions.

Figure 9 shows **Condition (2)**, which examines the dissimilarity of the payload data across byte positions. In the context of a fuzzy attack, the payload data of the current frame differ significantly from the payload data of other frames with the same message ID. Although an ideal approach would involve comparing the payload of the current frame with all the payloads in a normal dataset, such a method would require excessive computational resources. To circumvent this challenge, the present study pre-counts the number of “1” bits in each byte of the normal dataset and employs the highest counts as comparative thresholds. The quantity of bytes in the current payload data containing more “1” bits than those in the normal dataset serves as a measure of dissimilarity, and is designated as $BC_{cur}$.

As shown in Figure 9, the number of “1” bits in the current payload data is enumerated in terms of the respective byte positions. When the number of “1” bits in any byte of the current payload exceeds the predetermined maximum for that specific byte position in the normal dataset, $BC_{cur}$ increases by one for that particular position. The total number of bytes in the payload data of the current frame is denoted as $DLC_{cur}$. If $BC_{cur}\geq \frac{DLC_{cur}}{2}$, then it can be inferred that the current payload is substantially dissimilar from the payloads in the normal dataset for over half of the byte positions. Therefore, the current frame is classified as an attack because of its significant dissimilarity to normal frames. It is imperative to note that, according to Rule 2, a frame is identified as an attack only if both **Conditions (1) and (2)** are satisfied.

## 5. Experiments and Analysis

In this section, we describe the datasets used in our experiments involving conventional ML algorithms. We then present an evaluation method for the conventional IDS and the proposed IDS that incorporates a cross-check rule-based filter.

### 5.1. Dataset and Classifier Models

As shown in Table 1, the car hacking dataset [41] used in this study encompasses five distinct datasets. The attacks include a DoS attack involving the injection of ’0000’ CAN ID messages every 0.3 ms; a fuzzy attack in which completely random CAN ID and DATA values are injected every 0.5 ms; and spoofing attacks related to RPM/gear information, with specific CAN IDs being injected every 1 ms. Although the attack datasets comprised both attack and normal frames, the normal dataset was composed exclusively of normal frames. These datasets were constructed by recording CAN traffic via the OBD-II port in an actual vehicle (YF Sonata) while parked with the engine turned on during a message-injection attack. This attack involved the injection of counterfeit messages aimed at misleading the original ECUs, leading to vehicle malfunctions.

We partitioned the datasets into 70% and 30% subsets for training and testing, respectively. In addition, four conventional ML algorithms were employed, each specifically tailored for binary classification tasks. The conventional ML algorithms are presented as follows:

- **Random Forest (RF):** An ensemble learning method that operates by constructing multiple decision trees during training and outputting the mode of the classes for classification tasks.
- **k-Nearest Neighbors (kNN):** A simple algorithm that stores all available cases and classifies new cases based on a similarity measure.
- **Multilayer Perceptron (MLP):** A class of feedforward artificial neural network that contains layers of nodes and is used for pattern recognition.
- **Support Vector Machine (SVM):** A powerful and versatile machine learning algorithm used for both classification and regression tasks, which works by finding the best boundary that separates classes of data.

Similar to the DL models, these models have been employed in various security and privacy areas [42]. However, considering the limited capabilities of automotive devices, computing resources are often constrained for each vehicle [14]. Therefore, ML-based IDS are more appropriate for resource-constrained ECU than DL-based IDS.

### 5.2. Performance Metrics

The performance of the proposed method was evaluated using four performance metrics: Recall, Accuracy, Precision, and F1-score. These metrics are defined as follows:

- **Recall:** Often termed the true positive rate (TPR), this metric represents the proportion of correctly classified attack frames.
- **Accuracy:** This metric calculates the proportion of both true positives (TP) and true negatives (TN) among all evaluated cases.
- **Precision:** This metric calculates the proportion of TP to the total number of instances predicted as positive. In short, precision indicates the fraction of actual attacks within the predictions classified as attacks.
- **F1-score:** The F1-score is a metric that strikes a balance between precision and recall, often employed to assess classification performance, particularly in datasets with imbalanced class distributions.

The metrics are defined as follows:

(3) $$Recall=\frac{TP}{TP+FN}$$

(4) $$Accuracy=\frac{TP+TN}{TP+FP+FN+TN}$$

(5) $$Precision=\frac{TP}{TP+FP}$$

(6) $$F1-score=\frac{2\cdot Recall\cdot Precision}{Recall+Precision}$$

where TP represents the number of frames correctly classified as attacks, TN represent the number of frames correctly classified as normal, and false positives (FP) and false negatives (FN) represent the number of frames in which the model is incorrectly classified as attack or normal, respectively.

### 5.3. Experimental Results

**Figure 10:**
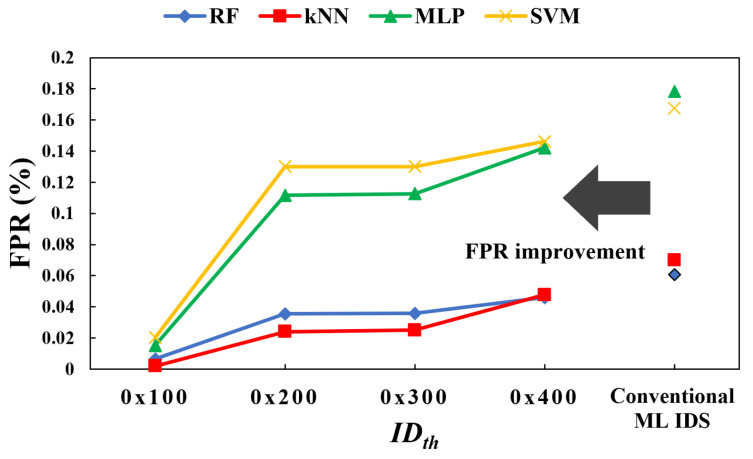
Improvement of false positive rate in DoS attack scenario according to $ID_{th}$.

Figure 10 shows the false positive rate (FPR) of the four ML-based IDS, highlighting how the performance of these systems is influenced by the threshold $ID_{th}$. According to the experimental results, if $ID_{th}$ is set to 0x400, the FPR increases to an average of approximately twelve times higher than when $ID_{th}$ is set to 0x100. Therefore, $ID_{th}$ should be appropriately determined based on the ID settings used in actual vehicles, as it influences the performance of our proposed cross-check system. The empirically determined threshold $ID_{th}$ was optimized through simulation and established at 0x100 for the performance comparison of the ML-based IDS variants conducted in this study.

**Figure 11:**
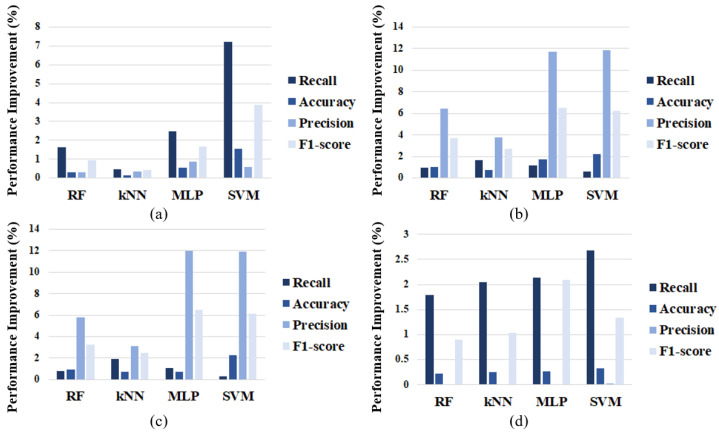
Performance improvement of ML-based IDS: (**a**) DoS attack; (**b**) gear spoofing attack; (**c**) RPM spoofing attack; (**d**) fuzzy attack.

Figure 11a–d show the rate of performance increase after implementing the proposed cross-check rule-based filters in terms of recall, accuracy, precision, and F1-score for DoS, gear spoofing, RPM spoofing, and fuzzy attacks, respectively. The performance improvement is defined as follows:

(7) $$\mathrm{Performance}\;\mathrm{Improvement}=\frac{M_{j}-M_{i}}{M_{i}}\times 100$$

where $M_{i}$ and $M_{j}$ represent the performance metrics before and after the application of the rule-based filters. As illustrated in the Figure 11, the proposed filters improved the performance of all ML-based IDS. In particular, the performances of the MLP and SVM models improved significantly. Overall, there was a tendency for recall to increase in DoS attacks and for precision to increase in gear and RPM spoofing attacks. However, in a fuzzy attack scenario, the performance improvement was lower than that observed in other types of attacks. This seems to be due to the higher complexity associated with fuzzy attacks compared to other types. Additionally, the algorithms used in the DoS and spoofing attack scenarios showed higher performance improvements due to the effective setting of attack frequency thresholds. In the fuzzy attack scenario, as evidenced by Figure 11d and described in Section 4, the approach to DBC can significantly influence performance improvement. The improvement in recall can be attributed to the first rule for fuzzy attacks, which treats message IDs not found in the normal dataset as attacks. Meanwhile, the precision remained largely unchanged due to reliance on the normal dataset. This can be attributed to the inability to accurately access the DBC, which results in legitimate IDs being mistakenly treated as attacks.

Table 2 enumerates the performance enhancements realized by applying the proposed rule-based filters evaluated across the four ML algorithms. Regardless of the specific evaluation metrics used, each algorithm demonstrated significant improvement in detection capabilities. The proposed rule-based filters are adaptable and capable of utilizing other predictive models to enhance attack detection performance in addition to the ML algorithms used in this study,. Additionally, these filters primarily execute comparison operations, which typically consume fewer computing resources than multiplication or division operations. In terms of time complexity, these filters can be expressed as either O(1) or O(n). However, in order for the proposed rule-based filters to cross-check the predictions of the IDS, the predictions must be made on a per-frame basis; for example, if the IDS is trained across multiple frames and only determines whether an attack is present or not, it would be challenging to apply these filters.

### 5.4. Comparison with Existing Works

We compared our proposed IDS architecture with other state-of-the-art IDS variants, as shown in Table 3. The most well-known DCNN model [10] shows almost perfect detection performance. However, it has a drawback in that it processes 29 CAN frames as a single input to determine the presence of an attack among these frames. This approach makes it difficult to identify exactly which frame contains the attack. GIDS [27] and NovelADS [29] are unsupervised learning-based IDS models capable of detecting unlearned attacks. However, similar to the DCNN model, they are limited in identifying precisely which frame contains an attack. CanNet [26] is a lightweight image classification network designed to detect anomalies in images generated from CAN data. The network evaluates the presence of an attack based on units of 16 CAN frames and uses a method that calculates the exact moment an attack occurs. However, the applicability of this network in other attack scenarios remains unclear, as experiments were conducted only on DoS attacks. The iForest-based IPS [30] offers the significant advantage of being able to detect attacks on a per-frame basis and remove the attacking frames. Nevertheless, this IPS has the limitation of relatively low detection performance compared to the other tested models.

The conventional ML algorithms we used for our experiments were trained on a per-frame basis; therefore, if the final decision from our cross-check system indicates an attack, it means that the current CAN frame has been identified as an attack. Table 3 shows the Random Forest model as a representative example of the ML algorithms used in the experiments. Compared to existing IDS models, it was observed that the Random Forest IDS enhanced with the proposed cross-check system exhibited superior detection performance for all types of attacks except for fuzzy attacks.

### 5.5. Discussion and Limitation

In the experiments conducted in this study, the proposed novel IDS architecture utilizing cross-check filters demonstrated improved performance across various types of attacks. However, our architecture has a significant limitation in detecting untrained types of attacks due to its reliance on supervised learning methods. Moreover, while the proposed filters take into account three typical types of attacks, it is possible that more varied types of attacks exist in the real world. To overcome this limitation, the proposed architecture needs to be applied to an unsupervised learning IDS to detect unknown attacks. In addition, it is necessary to collect and analyze different types of attacks in order to effectively cross-check and validate the system against various threats. Nevertheless, the proposed cross-check architecture remains effective against typical types of attacks for in-vehicle networks.

## 6. Conclusions

In this study, we have proposed a novel architecture for an IDS that incorporates rule-based filters to corroborate the classification outcomes of conventional ML algorithms. The experimental results demonstrate a marked enhancement in the detection performance of various ML-based IDS frameworks. This enhancement was particularly evident when the unique attributes of different attack scenarios were considered. Notably, the recall metric, which signifies the rate at which the attack frames were accurately classified, exceeded 99% after application of the proposed filters. However, these models inherently face challenges in identifying attack types that have not been included in their training owing to their foundation in supervised learning methods. Although further adjustments are required to address additional types of attack scenarios in real-world vehicular applications, this study provides a substantial foundation for the development of a comprehensive intrusion detection architecture.

In the future, we aim to refine our proposed rule-based filters to enhance their versatility across a broad spectrum of attack types. Currently, IDS models that analyze attacks on a per-frame basis tend to show lower performance compared to those that assess attacks over multiple frames. Therefore, our objective is to enhance our system by incorporating advanced filters into an unsupervised learning-based IDS. This improved system will be able to precisely identify the specific frame containing an attack while achieving superior overall detection performance. Moreover, the advanced filters will address various attacks not considered in this study. Every frame, whether normal or representing an attack, adheres to the CAN data format; specifically, attack frames can occur under four scenarios, namely, when the ID and the data field are each either fixed or random. By considering these cases, it would be possible further strengthen our cross-check system. 

## Figures and Tables

**Table 1:** Summary of the car hacking dataset.

Dataset	Normal Messages	Attacking Messages
DoS Attack	3,078,250	587,521
Spoofing Attack (Gear)	3,845,890	597,252
Spoofing Attack (RPM)	3,966,805	654,897
Fuzzy Attack	3,347,013	491,847
Normal	988,871	–

**Table 2:** Performance when applying the cross-check rule-based filters to conventional ML-based IDS.

Attack Type	ML Models	Recall (%)	Accuracy (%)	Precision (%)	F1-Score (%)
DoS attack	Random forest	98.391 → **100**	99.691 → **99.995**	99.679 → **99.966**	99.031 → **99.983**
	k-nearest neighbor (k = 10)	99.533 → **100**	99.866 → **99.998**	99.632 → **99.990**	99.583 → **99.995**
	MLP (2 hidden layers)	97.582 → **99.990**	99.462 → **99.986**	99.054 → **99.920**	98.312 → **99.955**
	Support vector machine	93.283 → **99.997**	98.452 → **99.983**	99.333 → **99.924**	96.213 → **99.960**
Gear attack	Random forest	99.083 → **100**	99.008 → **99.991**	93.877 → **99.936**	96.410 → **99.968**
	k-nearest neighbor (k = 10)	98.347 → **100**	99.263 → **99.987**	96.257 → **99.906**	97.291 → **99.953**
	MLP (2 hidden layers)	98.870 → **99.999**	98.264 → **99.993**	89.466 → **99.946**	93.857 → **99.972**
	Support vector machine	99.407 → **100**	97.827 → **99.993**	89.384 → **99.958**	94.130 → **99.979**
RPM attack	Random forest	99.224 → **100**	99.075 → **99.993**	94.498 → **99.952**	96.803 → **99.976**
	k-nearest neighbor (k = 10)	98.099 → **100**	99.296 → **99.997**	96.978 → **99.982**	97.535 → **99.991**
	MLP (2 hidden layers)	98.966 → **100**	99.296 → **99.997**	89.270 → **99.995**	93.868 → **99.998**
	Support vector machine	99.688 → **100**	97.806 → **99.997**	89.337 → **99.985**	94.229 → **99.993**
Fuzzy attack	Random forest	97.757 → **99.512**	99.539 → **99.762**	98.640 → **98.648**	98.197 → **99.079**
	k-nearest neighbor (k = 10)	97.568 → **99.571**	99.524 → **99.780**	98.710 → **98.724**	98.136 → **99.146**
	MLP (2 hidden layers)	97.290 → **99.368**	99.440 → **99.702**	98.291 → **98.307**	97.788 → **99.835**
	Support vector machine	96.740 → **99.331**	99.380 → **99.709**	98.389 → **98.412**	97.558 → **98.869**

**Table 3:** Comparison between the proposed IDS architecture and existing IDS models.

Attack Type	Models	Detection Units	Accuracy	Precision	Recall	F1-Score
DoS attack	DCNN [10]	29 CAN frames	99.97	100	99.89	99.95
	GIDS [27]	64 CAN frames	97.90	96.80	99.60	98.18
	NovelADS [29]	100 CAN frames	-	99.97	99.91	99.94
	CanNet [26]	16 CAN frames	99.66	100	99.77	99.88
	iForest [30]	per CAN frame	-	-	-	-
	**Proposed method**	**per CAN frame**	**99.99**	**99.96**	**100**	**99.98**
Fuzzy attack	DCNN [10]	29 CAN frames	99.97	100	99.89	99.95
	GIDS [27]	64 CAN frames	98.00	97.30	99.50	98.39
	NovelADS [29]	100 CAN frames	-	99.99	100	100
	CanNet [26]	16 CAN frames	-	-	-	-
	iForest [30]	per CAN frame	99.29	95.07	99.93	97.44
	**Proposed method**	**per CAN frame**	**99.76**	**98.64**	**99.51**	**99.07**
Gear attack	DCNN [10]	29 CAN frames	99.97	100	99.89	99.95
	GIDS [27]	64 CAN frames	96.20	98.10	96.50	97.29
	NovelADS [29]	100 CAN frames	-	99.89	99.93	99.91
	CanNet [26]	16 CAN frames	-	-	-	-
	iForest [30]	per CAN frame	99.24	94.79	100	97.33
	**Proposed method**	**per CAN frame**	**99.99**	**99.93**	**100**	**99.96**
RPM attack	DCNN [10]	29 CAN frames	99.97	100	99.89	99.95
	GIDS [27]	64 CAN frames	98.00	98.30	99.00	98.65
	NovelADS [29]	100 CAN frames	-	99.91	99.90	99.91
	CanNet [26]	16 CAN frames	-	-	-	-
	iForest [30]	per CAN frame	99.85	98.97	100	99.48
	**Proposed method**	**per CAN frame**	**99.99**	**100**	**99.95**	**99.97**

## Data Availability

https://ocslab.hksecurity.net/Datasets/car-hacking-dataset (accessed on 28 March 2024).
